# Evaluation of the Efficacy of a New Commercially Available Inactivated Vaccine Against Ovine Enzootic Abortion

**DOI:** 10.3389/fvets.2020.00593

**Published:** 2020-09-04

**Authors:** Carlos Montbrau, Mireia Fontseca, Ricard March, Marta Sitja, Julio Benavides, Nieves Ortega, María Rosa Caro, Jesús Salinas

**Affiliations:** ^1^Hipra Scientific, S.L.U., Girona, Spain; ^2^Instituto de Ganadería de Montaña (CSIC-Universidad de León), León, Spain; ^3^Department of Animal Health, Faculty of Veterinary, Mare Nostrum International University of Murcia, Murcia, Spain

**Keywords:** *Chlamydia abortus*, ovine enzootic abortion, sheep, vaccine, abortion

## Abstract

Ovine enzootic abortion (OEA), caused by *Chlamydia abortus*, is an economically important disease in many countries. Inactivated vaccines have been used for many years as they induce immunity in sheep, although outbreaks of abortions have been described in vaccinated flocks. In addition, there is a commercially available live attenuated vaccine that provides good protective results. Recently however, reports question the attenuation of this vaccine and associate it with the appearance of outbreaks of OEA in vaccinated flocks. In the present study, a recently commercialized inactivated vaccine (INMEVA®; Laboratorios Hipra S.A., Amer, Spain) has been evaluated using mouse and sheep experimental models. In the mouse models (non-pregnant and pregnant models), the efficacy of INMEVA vaccine has been compared to an unvaccinated control group and to an experimental inactivated vaccine considered as a positive protection control (UMU vaccine). In the non- pregnant model, the UMU vaccine was more effective than the INMEVA vaccine regarding the impact on body weight or the presence of *C. abortus* in the liver, but both vaccinated groups (UMU and INMEVA) had significantly lower *C. abortus* in the liver compared to the control group. In the pregnant model in terms of reproductive failures, pups per mouse or the presence of *C. abortus* in the liver or uterus, no significant differences were found between both vaccines, inducing protection compared to the control group. In the ovine pregnant model, where INMEVA vaccine was compared only to an unvaccinated group, the results indicate that this new commercial vaccine is safe and provides a suitable level of protection against an experimental challenge with *C. abortus*. A 75% reduction in reproductive disorders, 55% reduction in animals with *C. abortus* shedding on day of parturition/abortion, and a significant reduction of the average amount of chlamydial shedding from parturition/abortion over the next 21 days was observed, in relation to the infected control group. The results suggest that this vaccine is adequate for the control and prevention of OEA; however, future studies are necessary to elucidate the type of protective immune response that it induces.

## Introduction

Ovine enzootic abortion (OEA), caused by *Chlamydia abortus*, an obligate intracellular Gram-negative bacterium belonging to the *Chlamydiaceae* family, is an economically important disease in many countries. In fact, OEA is one of the major causes of small ruminant abortion and is found throughout the world (including North America, Europe and Africa), but not in Oceania (Australia and New Zealand) (1). In addition, it has been described that *C. abortus* can cause miscarriages in women (2) and atypical pneumonia in humans (3) as a zoonotic agent. The infection results in a purulent placentitis, leading to reproductive failures such as late-term abortion, premature lambing with neonatal death or the birth of weak lambs (4). The disease spreads easily in infected flocks, particularly at lambing time when ewes shed large amounts of *C. abortus* via vaginal discharges at abortion or parturition (placenta and fetuses). On the other hand, this infection will provide an effective immune response in OEA-affected ewes, protecting from future *C. abortus*-induced abortions. They could, however, shed the bacteria in subsequent estrus or parturition, maintaining the risk of infection in the flock (5). The ingestion of contaminated material or inhaling aerosols can infect susceptible animals (6). After an infection of a naïve ewe it is believed that *C. abortus* may remains in a latent state in lymphoid tissue, controlled by cytokines like IFN-γ (7), and may not show clinical signs until the last weeks of the next gestation, leading to potential reproductive failure. The reproductive failure rate in an endemically infected flock is around 10%, whereas in a newly infected naïve flock it is around 30%, up to 60% in goat herds (4).

In the last 60 years, different inactivated vaccines prepared from egg grown or cell cultures have been commercialized for preventing OEA. Nevertheless, the efficacy of these vaccines has been questioned, as in some cases, outbreaks have been described in vaccinated flocks (8–10). Inactivated vaccines can reduce the incidence of abortions and the shedding of *C. abortus* at parturition, but they do not prevent shedding completely. This fact can cause endemic cycles of infection, with serious consequences for the OEA epidemiology (11). The outcome of this lack of efficacy is that most of these vaccines have been removed from the market (10). To overcome this problem, live temperature-sensitive attenuated vaccines have been developed, represented by the 1B strain derived from the wild-type AB7 strain after nitrosoguanidine (NTG) treatment (12). This vaccine is commercially available and good results in protecting against *C. abortus*-induced abortion in field trials have been reported (6, 13). However, given the zoonotic capacity of the pathogen, as well as the recently reported occurrence of abortive outbreaks in vaccinated flocks, in which the vaccine strain was involved, this attenuated vaccine raises safety risks. Indeed, after identification by PCR-RFLP of several single nucleotide polymorphisms (SNPs) exclusive to the vaccine strain 1B (14), it was possible to discriminate between wild-type field strains from the vaccine strain, and the vaccine strain was identified in abortions of vaccinated animals (15–19), where some unvaccinated animals suffered abortions caused by this vaccine strain. In addition, in a recent study, Longbottom et al. (19) question the true attenuation of the vaccine strain and have concluded that the protection of the 1B vaccine is unlikely to be due to the NTG induced SNPs and is more likely caused by the administration of high doses of *C. abortus* elementary bodies that stimulate protective immunity, similar to a natural infection.

Other vaccine approaches, such as subcellular vaccines or DNA vaccines, have recently been studied. The major outer membrane protein (MOMP) has been the main focus of subcellular vaccines, where protection has been described by using its native oligomer form (20). Vaccines comprising the chlamydial outer membrane complex provided good protection against OEA (21). However, the large quantities of Chlamydiae needed for vaccine preparation have made it economically unfeasible for an ovine vaccine (22). Studies conducted to assess the efficacy of recombinant MOMP against *C. abortus* experimental infection have been discouraging (23). In a recent study, O'Neill et al. (24) demonstrated 50% efficacy when two recombinant proteins were administered together in a pregnant ovine model. Nonetheless, bacterial shedding was not reduced. Finally, all attempts to get a DNA vaccine against OEA have failed to induce a protective immune response [reviewed in (25)]. Despite the different approaches in the design of vaccines against *C. abortus*, there is still a lack of safe and efficacious vaccines in terms of shedding and reproductive disorders.

The mouse models have proven to be a well-controlled experimental animal model for assessing the efficacy of experimental or commercial vaccines (26–29). In fact, it has been shown that intraperitoneal inoculation of *C. abortus* between 7 and 11 days of gestation in mice induces colonization of the placenta, leading to abortion and the shedding of *C. abortus* (30). Similarly to the experimentally-induced or natural disease in ewes. In addition, this model has been used to test different adjuvant and vaccine-production procedures for the development of new inactivated vaccines against OEA (28). In that previous study, the efficacy of a new experimental inactivated vaccine was demonstrated. In this context, the first aim of this study was to test a new inactivated vaccine against OEA that has recently been commercialized in different European countries under the name INMEVA® (Laboratorios HIPRA, S.A., Amer, Spain). To do that, a pregnant mouse model challenged with *C. abortus* at mid gestation was used to assess INMEVA and compare it to the efficacious experimental inactivated vaccine. However, as it was necessary to test this vaccine on the target animals, the second aim was to evaluate the efficacy of the new commercial inactivated vaccine in a pregnant sheep model.

## Materials and Methods

### Mice and Housing

Seven- to eight-week-old female Swiss OF1 mice were purchased from Harlan UK Limited (Bicester, UK). All of them were free of common bacterial and viral pathogens based on the results of routine screening procedures conducted by the suppliers. The food and water were offered *ad libitum* and mice were kept in cages in an environmentally controlled room. The study was conducted at the animal facilities of the University of Murcia (UMU, Murcia, Spain). All handling practices were endorsed by the local government and followed the recommendations of Directive 2010/63/EU of the European Parliament and of the Council on the protection of animals used for scientific purposes and the Bioethical Committee of the University of Murcia, Spain (approval number A13150202; date of approval 24 February 2015). All animals enrolled in the study were handled following strictly the good clinical practices and all efforts were taken to minimize suffering.

### Ewes and Housing

Eighty primiparous ewes of the breed “Churra” were purchased from a flock in Castilla y León (north-western Spain) with no known previous cases of *Chlamydia abortus* and which had not been administered any previous vaccine against OEA. These ewes were serologically screened for *C. abortus* (using ID Screen® Chlamydophila abortus Indirect Multi-species, ID.VET, France), *Salmonella* Abortusovis (using an in-house ELISA test, Hipra, Spain), *Brucella spp*. (using ID Screen® Brucellosis Serum Indirect Multi-species, ID-VET, France), *Toxoplasma gondii* (using ID Screen® Toxoplasmosis Indirect Multi-species, ID-VET, France), *Coxiella burnetii* (using ID Screen® Q Fever Indirect Multi-species, ID-VET, France), and *Maedi-Visna* virus (using ID Screen® MVV/CAEV Indirect, ID-VET, France) before being included in the study. The group size used was determined to contain at least 15 pregnant ewes. This group size (*n* = 15) was based upon a 75% reduction in reproductive disorders caused by *C. abortus* (such as abortions in late pregnancy, premature lambing or the birth of weak lambs) after challenge in vaccinated vs. control animals. The estimated percentage of reproductive disorders of ewes becoming infected with *C. abortus* in the control group was 65%. Consequently, a 16.25% of reproductive disorders was estimated in the vaccinated group. The required number of animals (*n* = 15) was obtained using the Ene 3.0 software (Servei d'Estadística Aplicada, Universitat Autonoma de Barcelona and the Biometric Department of GlaxoSmithKline, Bellaterra, Spain), considering a power (1-β) of 0.80 and α of 0.05. Sixty ewes were then randomly divided into two experimental groups of 30 animals (vaccinated and control group). An additional group of 20 animals was included as a sentinel group (not infected). Ewes were randomly divided into three experimental groups (A, B, or C). Distribution of these ewes into 3 groups was performed by sorting those ewes based on their body weight 2 days prior to vaccination. Five cohorts of 16 ewes were generated; Cohort 1 contained the 16 ewes with the lowest body weight, whereas cohort 5 contained the 16 ewes with the highest body weight. A random number was generated using computer software (Excel 2013, Microsoft Corp., Redmond, WA). The six ewes with the lowest random numbers of each cohort were assigned to group A. The six ewes with the highest random numbers of each cohort were assigned to Group B. The remaining 4 ewes per cohort, with the intermediate random number were assigned to Group C. Consequently, 30 ewes were assigned to the vaccinated group (39.0 ± 0.89 kg), another 30 ewes were assigned to the control group (39.19 ± 0.91 kg) and 20 ewes were assigned to the sentinel group (39.4 ± 0.93 kg). Ewes were uniquely identified with paired, sequentially numbered ear tags, 1 in each ear. The study was conducted in the Instituto de Ganadería de Montaña (CSIC-Universidad de León, León, Spain). All sheep were handle following the recommendations of Directive 2010/63/EU of the European Parliament and of the Council on the protection of animals used for scientific purposes and the CSIC Animal Experimentation Committee (ref. OH/406-2016). This phase was conducted over a 9-month period from August to April on an experimental farm (Grulleros, Spain), where the average low and high ambient temperatures were 4 and 18°C, respectively. Relative humidity ranged from 44% (average daily low) to 89% (average daily high). During the vaccination phase, all ewes were housed together in a single pen with straw bedding and physically separated from other animals. Bedding material was replaced weekly. Food and water were offered *ad libitum* during the study.

Five and 2 weeks before mating, all animals received 2 mL of vaccine or PBS subcutaneously. Then, ewes were estrus-synchronized through vaginal sponges with medroxyprogesterone acetate (Esponjavet, HIPRA, Spain) for 14 days and subsequent intramuscular inoculation of mare serum gonadotropin (Oviser, HIPRA, Spain). All animals were then mated with pure-breed Churra rams during 2 days, after that the ewes were separated from the rams; all those rams were serologically negative to *C. abortus*. Gestation and fetal viability were diagnosed by ultrasound scanning (US) on day 45 post-mating. Non-pregnant animals were withdrawn from the study. A total of 50 ewes were pregnant: 19 vaccinated, 21 control, and 10 sentinels. All these pregnant ewes were enrolled in the challenge phase. During this phase, infected ewes were housed together in four pens of 10 animals each, to minimize the stress of handling during the sampling and to facilitate the detection of abortions. The sentinel group was housed in a separate pen. All these pens had straw bedding and were physically separated from other animals. New straw bedding was added approximately weekly. Food and water were offered *ad libitum* during the study.

### Vaccines and Inoculum

The vaccine evaluated in the present study was an inactivated vaccine of *Salmonella enterica* subsp. *enterica* serovar Abortusovis and *Chlamydia abortus* (INMEVA® Laboratorios Hipra S.A., Amer, Spain). This vaccine contains the A22 strain of *C. abortus* inactivated by a method involving nucleic acids but not surface proteins. This vaccine has a mixture of aluminum hydroxide and diethylaminoethyl (DEAE) dextran as an adjuvant.

The abortion-causing *C. abortus* AB7 strain (31) was used to prepare the homologous vaccine (experimental vaccine UMU) in the experiment 1 developed in the murine model. This strain was also used to prepare the inoculums of murine models and ovine model. The bacterial strain was inoculated into yolk sacs of developing chicken embryos. Inoculum titration was performed by enumerating the inclusion-forming units (IFU) in McCoy cells, and then the standardized aliquots were frozen at −80°C until use. To perform the infection, freshly thawed vials were diluted with sterile phosphate-buffered saline (PBS) 0.1M pH 7.2 to final concentrations of 2 × 10^5^ IFUs/mL for non-pregnant mice, 5 × 10^4^ IFUs/mL for pregnant mice or 1.5 × 10^6^ IFUs/mL for ewe challenge. Challenged mice received 0, 2 mL intraperitoneally and pregnant ewes were challenged subcutaneously with 2 mL. The homologous vaccine (UMU) using the AB7 strain was developed as previously described by Caro et al. (28). This experimental vaccine was inactivated by binary ethylenimide (BEI) and adjuvated with the semi-purified derivate of saponin QS-21. This UMU experimental vaccine has been previously tested showing that it induces excellent protective results in different mouse models [reviewed in (29)], so it was used as a positive protection control in the murine models.

### Study Design of Experiment 1 (Mice)

**Figure 1:**
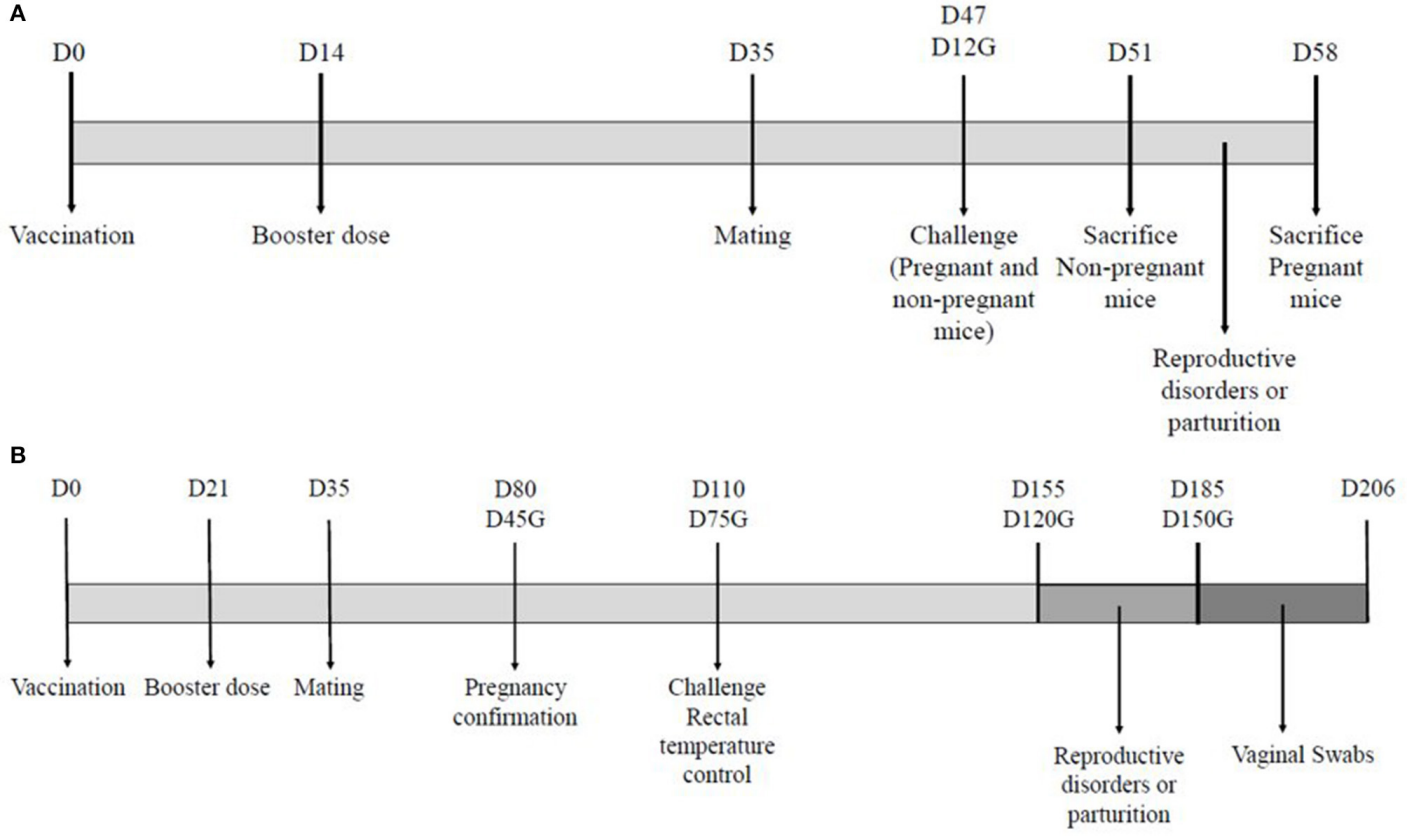
Experimental designs for the mouse model **(A)** and the ovine model **(B)**. The diagrams illustrate the days (D) of the different interventions on the animals used in both experiments. The day of gestation is indicated for pregnant animals (G).

All mice were enrolled in the study 7 days before vaccination. Mice were randomly distributed in four groups. Group A (*n* = 20) was immunized subcutaneously with the new inactivated vaccine of *C. abortus* (INMEVA). Group B (*n* = 20) was immunized with the homologous inactivated vaccine of *C. abortus* (UMU). Group C (*n* = 20) was mock-vaccinated with PBS as a control group. Group D (*n* = 10) was monitored as a sentinel group. Animals of groups A, B, and C received 0.2 mL of vaccine or PBS subcutaneously in the back on the right of the midline on day 0 and 14 days later on the left of the midline. (Figure 1A). General appearance and possible clinical signs were monitored daily. Once a week the injection sites were palpated to detect signs of pain and examine for swelling. Three weeks post-boost (day 35 of study), mice were mated, and the detection of a vaginal plug was considered as day 0 of gestation. All pregnant mice and six non-pregnant mice from groups A, five non- pregnant mice of group B and five non- pregnant mice of group C were infected 33 days after administering the post-boost (day 47 of study). Consequently, in pregnant animals, challenge was conducted at 12 days of gestation (Figure 1A). All mice were monitored to assess demeanor throughout the entire study.

After challenge, non-pregnant mice were monitored daily to evaluate mortality, and body weight was recorded on the day of challenge (D47) and the day of necropsy (D51). Pregnant mice were monitored daily for mortality, abortions and the number of live offspring per litter from the experimental infection to the day of necropsy (11 days after challenge). On the day of necropsy all animals were euthanized and sampled to detect the presence of *C. abortus* from liver and from the uterine horn in the case of the pregnant animals.

### Study Design of Experiment 2 (Ewes)

All ewes were enrolled in the study 14 days before the vaccination. The ewes of group A (*n* = 30) were immunized with the new inactivated vaccine INMEVA, whereas the other two groups, control and sentinel (B and C; *n* = 30 and 20, respectively), were administered PBS (Figure 1B).

During the vaccination phase, from vaccination until diagnosis of gestation, general clinical signs were monitored daily. Subsequently, all 40 pregnant ewes of groups A (*n* = 19) and B (*n* = 21) were challenged subcutaneously on day 75 of gestation. Group C (*n* = 10) was not infected, remaining as a sentinel group.

During the challenge phase, visual examinations were conducted to assess ewe demeanor and reproductive incidences (abortions in late pregnancy, premature lambing or the birth of weak lambs, etc.), which were recorded daily from challenge until 1 month after parturition/abortion. In the event of an abortion, vaginal swabs, lung samples from the fetus, or dead lamb, and placenta samples were collected. Furthermore, to assess the shedding of *C. abortus*, vaginal swabs were collected from all infected ewes on the day of challenge, the day of abortion or parturition and then three times a week during the 21 days following the day of abortion or parturition. All those samples collected from aborted fetuses or dead lambs, and vaginal swabs were analyzed by qPCR, as described in the following section. Rectal temperatures of all animals were recorded daily from 3 days before challenge to 5 days after challenge. Body weight of lambs was recorded at parturition and then 4, 7, 11, 14, 18, 21, 25, and 28 days post-partum. If an abortion occurred, macroscopic evaluation of the placenta and fetuses was carried out. Next, samples from placenta and fetal brain, lung, and liver were fixed in 10% buffered formalin and further processed for conventional histopathological evaluation and labeling of chlamydia. Immunohistochemical detection of the chlamydial antigen was carried out following the same previously published technique (32). The personnel involved in the animal experimental procedures, who gathered data on different parameters, were not aware of the treatment received by each individual ewe because ewes and treatments were randomized by a treatment dispenser who was not involved in gathering data.

### Isolation of *C. abortus*

In mice experimental design, the evaluation of the infection was performed by counting the IFUs of the liver and spleen isolated on McCoy cell monolayers, based on the method previously described by Buendia et al. (33). The number of inclusions in McCoy cells was counted under fluorescent microscopy to calculate the number of IFUs per gram of tissue. The analysis of the persistence of *C. abortus* in the uterus was conducted taking a complete uterine horn, which had at least four sites of previous placental attachment, and following the procedure used for the other tissues, to calculate the number of IFUs per uterine horn. This method has a detection limit of 66.6 IFUs per horn.

### *C. abortus* Quantitative PCR

Referring to the ovine experiment, vaginal swabs were resuspended in 1.5 mL of sterile PBS and tissue samples diluted 1/5 w/v with sterile PBS and homogenized. The DNEasy Blood and Tissue Kit (Qiagen, Las Rozas, Madrid) was used to process the samples, following the instructions of the manufacturer. All samples of DNA extracted was analyzed using a TaqMan PCR for quantification of *C. abortus*. The *omp A* gene of *C. abortus* was amplified following the method described by Pantchev et al. (34). Real-time PCR was performed in duplicate using the QuantiFast Pathogen IC PCR kit (Qiagen) as universal PCR Master Mix in a final volume of 25 μL per reaction. The mixture included 5 μl of sample DNA, 1 μL of internal control assay, 1 μL of internal control DNA, each primer (Cpa OMP1-F and Cpa OMP1-R) at the final concentration of 0.9 and 0.2 μM of Cpa OMP1 probe. Amplification was carried out on a Light Cycler 480 (Roche) thermocycler, using the following cycling parameters: 95°C for 5 min (single denaturation step), 45 cycles of 95°C for 15 s and 60°C for 1 min (annealing and extension). The fluorescence data was collected using the ROX and FAM filters during the 60°C annealing step.

A 10-fold serial dilution of inoculum of *C. abortus* (10^8^ IFU/mL) were made and the extraction of DNA was done in each diluted sample using the same protocol as the samples to create a standard curve to estimate the number of genome copies of *C. abortus* in each sample.

### Calculation and Statistical Analysis

Body weights per group of the non- pregnant mice on day 47 (challenge day) and day 51 were compared per day using PROC MIXED in SAS (SAS Institute Inc., Cary, NC), treating a day as a repeated measure. The model included the fixed effects of day, treatment, day-by-treatment interaction, and the random effect of animal within the treatment. Compound symmetry was selected as the variance-covariance matrix structure on the basis of best fit according to Schwarz's Bayesian information criterion. Using these data, the percentage of body weight gain per animal was calculated as the difference between final body weight (D51) minus the body weight on challenge day (D47) divided by the body weight of challenge day. These percentages from the different groups were compared using ANOVA in SAS. The data of amount of *C. abortus* (IFUs/mL) isolated in the liver samples were log-transformed to fulfill normality assumptions. The amount of *C. abortus* (log IFU/mL) of each group was compared using ANOVA in SAS.

In the pregnant mice trial, the percentage of reproductive disorders caused by *C. abortus* and the percentage of liver and uterus samples with *C. abortus* per group were compared using the chi-squared test within PROC FREQ in SAS. The number of pups per litter of each group was compared using ANOVA in SAS. The amount of *C. abortus* isolated in the liver and uterus samples was compared using the Kruskal-Wallis test within PROC NPAR1WAY of SAS; therefore, data were not parametric. Dunn's test was used to compare the pairs.

In the ovine experiment, the number of reproductive incidents (abortions in late pregnancy, premature lambing or the birth of weak lambs with early mortality) caused by the experimental infection of *C. abortus* (confirmed by PCR) per day, from challenge to the end of the study, was analyzed using the Fisher's exact (2-sided) test within PROC FREQ in SAS.

Vaginal swab samples collected from abortion or parturition to 21 days after were used to determine the degree and duration of *C. abortus* shedding. Shedding of *C. abortus* detected on the day of abortion/parturition per group were used to compare groups. After parturition or abortion, at least 9 further samples were collected per animal, on Monday, Wednesday and Friday. Thus, depending on the day of parturition or abortion, samples were collected on different days. To summarize these data, an average per animal was calculated at these different time intervals: D0–D3, D4–D6, D7–D9, D10–D12, D13–D15, D16–D18, and D19–D21 post-partum/abortion. The area under the curve (AUC) of shedding of *C. abortus* from abortion or parturition to 21 days after was calculated for each ewe using the data gathered from this period. One value per ewe was obtained, estimating the total amount of shedding (expressed in number of genome copies of *C. abortus* per sample) from abortion or parturition to 21 days after. The shedding of *C. abortus* detected on the day of abortion/parturition per group were also used to compare groups. The amount of *C. abortus* isolated in the vaginal swab samples on the day of abortion/parturition, at different intervals detailed above and during the 21 days after abortion/parturition (AUC), were compared using the Kruskal-Wallis test within PROC NPAR1WAY of SAS; therefore, data were not parametric Dunn's test was used to compare the pairs. The percentage of samples positive by PCR against *C. abortus* on the day of abortion/parturition and from abortion or parturition to 21 days after per group was compared using the Fisher's exact (2-sided) test within PROC FREQ in SAS.

Rectal temperature data around the day of challenge per group were compared using PROC MIXED in SAS (SAS Institute Inc., Cary, NC), treating a day as a repeated measure. The model included the fixed effects of day, treatment, day-by-treatment interaction, and the random effect of animal within treatment. Compound symmetry was selected as the variance-covariance matrix structure on the basis of best fit according to Schwarz's Bayesian information criterion. A rectal temperature in ewes between 39.0 and 40.0°C (35) was considered normal. The temperature data were converted into binomial, normal values (<40.0°C) or abnormal values (>40.0°C). These data per day were analyzed using the chi-squared test within PROC FREQ in SAS.

The body weight of lambs per group was compared using the Student's *t-*test in SAS per day. All values reported are least squares means. Significance was declared at *P* < 0.05.

## Results

### Results of Experiment 1 (Mice)

#### Adverse Reactions to Vaccines

No clinical signs or macroscopic lesions were observed in mice vaccinated with the experimental UMU vaccine or PBS control groups. In contrast, the INMEVA vaccinated mice showed swelling measuring 0.4–0.9 cm at 1–2 weeks after the primary and secondary administration. These local reactions disappeared without treatment 3 weeks after vaccination. However, no animal showed signs of pain, like squeaking, vocalizing or struggling, on palpation.

#### Effect of Vaccines on Protection in a Non-pregnant Mouse Model

Immunized non-pregnant mice were challenged on day 33 post-boost administration (day 47 of study) with the AB7 strain of *C. abortus*. The infected control group showed high morbidity, with acute signs of disease such as weakness, ruffled fur, lethargy and a substantial reduction in body weight, while none of the vaccinated animals showed clinical signs. However, the UMU immunized mice had significantly (*P* < 0.05) higher body weight and body weight gain compared to immunized with the INMEVA vaccine and compared to the control group at day 4 post-infection (Table 1). A lower number of bacteria in the liver was isolated in the mice vaccinated with UMU, showing significant differences (*P* < 0.05) compared to INMEVA and control groups. Furthermore, animals vaccinated with INMEVA had significantly (*P* < 0.05) lower *C. abortus* isolation from liver than those of the control group (Table 1).

**Table 1:** Average body weight on day 47 (challenge day) and 51 of study, percentage of difference in bodyweight from day 47 to 51 and *C. abortus* isolation from liver samples (log IFU/g of tissue) of non-pregnant challenged mice of each group.

		**INMEVA**	**UMU**	**Control group**
Body weight	D47 (Challenge day)	29.8 ± 0.73^b^	29.6 ± 0.68^b^	32.1 ± 0.82^a^
	D51	27.7 ± 0.81^b^	30.2 ± 0.67^a^	28.3 ± 0.53^b^
Body weight gain (%)		−7.21 ± 1.42^b^	1.97 ± 0.57^a^	−11.8 ± 1.71^b^
*C. abortus* isolation	Percentage of animals with *C. abortus*	6/6 (100%)	5/5 (100%)	5/5 (100%)
	Log_10_ IFUs/g (Mean ± SEM)	5.78 ± 0.29^b^	4.26 ± 0.26^c^	6.83 ± 0.07^a^

*Different superscript letters per row (a, b, c) indicate significant differences (P < 0.05) between groups*.

#### Effect of Vaccines on Protection in a Pregnant Mouse Model

Immunized pregnant mice were challenged on day 33 post-boost administration (day 12 of gestation, day 47 of study) under the same conditions as non-pregnant mice, and animals were monitored to evaluate the reproductive disorders caused by *C. abortus* as well as the presence of the microorganism in the liver and uterus on day 11 post-challenge. None of the UMU immunized mice showed abortions unlike the 25% of the INMEVA immunized mice, but no significant differences were noticed between these two vaccinated groups. On the other hand, 89% of the control mice had abortions, which was significantly (*P* < 0.05) higher compared to vaccinated or sentinel groups (Table 2), showing significant differences between vaccinated and non-vaccinated groups. Similarly, the number of pups per mouse was significantly (*P* < 0.05) higher in the vaccinated than in the control group. In addition, the percentage of presence of *C. abortus* as well as the load of microorganisms from liver and uterus collected was significantly lower in vaccinated animals than in the control group. No significant differences were observed between the two vaccinated groups (Table 2).

**Table 2:** Presence of reproductive disorders caused by *C. abortus*, average number of pups per mice, the percentage of liver and uterus samples with presence of *C. abortus* and the average titration of *C. abortus* from liver and uterus samples (log IFU/g of tissue) of pregnant mice per group.

		**INMEVA**	**UMU**	**Control**	**Sentinel**
Reproductive disorders caused by *C. abortus*		2/8 (25%)^b^	0/11 (0%)^b^	8/9 (89%)^a^	0/9 (0%)^b^
Number of pups per mice (Mean ± SEM)		8.63 ± 1.78^a^	12.91 ± 1.11^a^	1.89 ± 1.11^b^	12.11 ± 1.07^a^
*C. abortus* isolation (liver)	Percentage of animals with *C. abortus*	2/8 (25%)^a^	2/11 (18%)^a^	8/9 (89%)^b^	0/9 (0%)^a^
	Log_10_ IFUs/g (Mean ± SEM)	0.79 ± 0.52^b^	0.47 ± 0.32^b^	3.17 ± 0.44^a^	0.00^b^
*C. abortus* isolation (uterus)	Percentage of animals with *C. abortus*	2/8 (25%)^a^	0/11 (0%)^a^	8/9 (89%)^b^	0/9 (0%)^a^
	Log_10_ IFUs/g (Mean ± SEM)	0.94 ± 0.62^b^	0.00^b^	3.90 ± 0.27^a^	0.00^b^

*Different superscript letters per row (a, b) indicate significant differences (P < 0.05) between groups*.

### Results of Experiment 2 (Ewes)

#### Safety After Vaccination

After vaccination, clinical signs were evaluated and animal behavior was considered normal during the monitoring period. The daily rectal temperatures of vaccinated and control sheep were similar after vaccination and no increase was detected in any group (data not shown). No alterations were observed in vaccinated or control animals during the gestation period from time of vaccination to the ultrasound detection of gestation and fetal viability on day 45 post-mating. No significant differences in terms of percentage of gestation were observed among groups, where 19 out of the 30 vaccinated ewes, 21 out of the 30 control ewes and 10 out of the 20 sentinel ewes became pregnant after mating.

#### Reproductive Incidences Related to *C. abortus* and Shedding After Challenge

Thirteen days after challenge, a vaccinated animal suffered a traumatic abortion, as this animal was accidentally caught at the trough for several hours. Samples from the fetus and placenta were collected and no lesions suggesting infectious abortion were found. Moreover, the samples tested negative to *C. abortus* by PCR, confirming that this abortion was unrelated to the infection. Consequently, this animal was eliminated from the study. Therefore, the vaccine efficacy was tested using 18 vaccinated animals.

**Figure 2:**
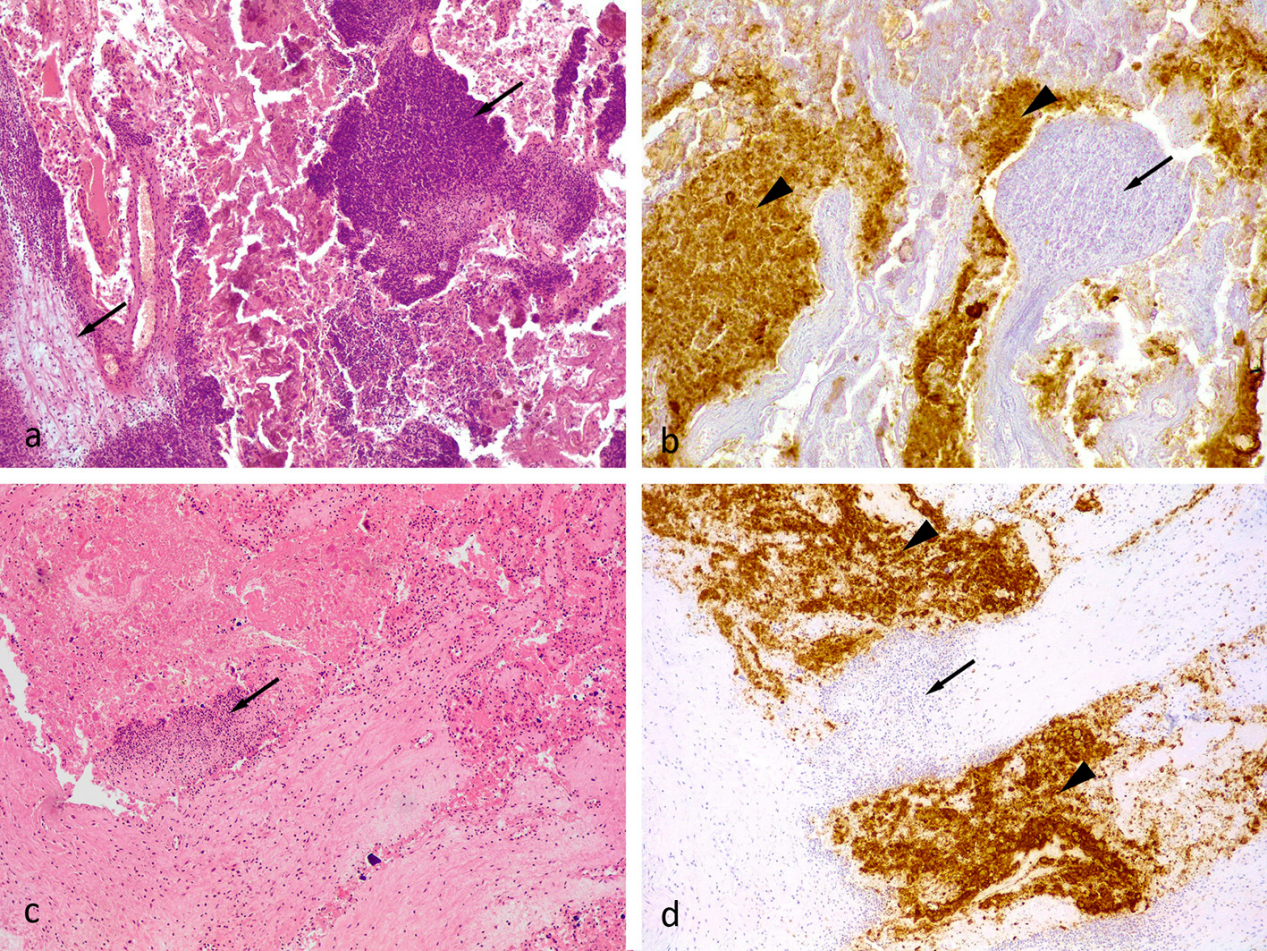
**(a)** Ovine placenta, midgestation, from an unvaccinated aborted fetus (positive control). Severe multifocal necro-suppurative placentitis. The inflammatory cell infiltrate, primarily neutrophils and cellular debris, was in the villous projections of the maternal caruncle (arrows). HE. 10x. **(b)** The Chlamydial antigen (brown pigment) was present within necrotic villous projections of the fetal cotyledons (arrowhead). There was little to no labeling within the inflammatory cell infiltrate present within the maternal caruncles (arrow). Semi-serial section of **(a)**. Chlamydial antigen IHC. 10x. **(c)** Placental sample from a stillborn unvaccinated animal (positive control). Mild suppurative placentitis as denoted by a single focus of inflammatory cells, primarily neutrophils, within a villous projection of the maternal caruncle (arrow). HE. 10x. **(d)** Semi-serial section of **(c)**. Similar to figure **(b)**, the chlamydial antigen (brown pigment) was present mostly in the cellular debris within the fetal cotyledon (arrowhead). There was little to no labeling within the inflammatory cell infiltrate within the tissue of the maternal caruncles (arrow). Chlamydial antigen IHC. 10x.

Two control group abortions were reported during the gestation period. Both ewes aborted two fetuses and all samples collected from these abortions (vaginal swabs, placenta and fetus samples) were positive to *C. abortus* by PCR. A severe subacute and multifocal to coalescent suppurative and necrotic placentitis was observed involving both cotyledons and intercotyledonary membranes (Figure 2A). Immunohistochemical labeling showed abundant chlamydial antigen within the fetal necrotic villi in these samples (Figure 2B). Afterward, three ewes from the control group had stillbirths in a premature parturition, where all samples collected were also positive to *C. abortus* by PCR. Histological lesions in these cases were milder than those found in the samples from abortions and characterized by a moderate multifocal suppurative placentitis, mainly affecting the base of the placentomes. These lesions appeared as discrete foci of inflammatory cells, mainly neutrophils, within the caruncular villi (Figure 2C). Immunohistochemical labeling showed focal accumulation of chlamydial antigen in cotyledonary villi adjacent to the foci of inflammatory cells at the caruncular villi (Figure 2D)

After that, four control and two vaccinated ewes gave birth to weak lambs, which died between 1 and 8 days after parturition and all samples collected were also positive to *C. abortus* by PCR, confirming the involvement of *C. abortus* in the perinatal death of the lambs. No other reproductive incidences related to *C. abortus* were noticed during the study. Consequently, the percentage of reproductive incidences was significantly (*P* < 0.05) greater in the control group compared to vaccinated and sentinel ewes (Table 3).

**Table 3:** Presence of reproductive disorders caused by *C. abortus*, the percentage of vaginal swab samples with presence of *C. abortus* and the average titration of *C. abortus* from vaginal swab samples (numbers of genome copies of *C. abortus* per sample) of pregnant ewes per group.

		**Vaccinated**	**Control**
Reproductive disorders caused *C. abortus*		2/18 (11%)^b^	9/21 (43%)^a^
*C. abortus* at parturition/abortion	Percentage of animals with *C. abortus*	7/18 (39%)^b^	18/21 (86%)^a^
	Log_10_ genome copies	2.03 ± 0.62^b^	5.11 ± 0.51^a^
*C. abortus* from parturition/abortion to 21 days after	Percentage of samples with *C. abortus*	35/179 (20%)^b^	131/210 (62%)^a^
	Log_10_ genome copies (AUC)	2.46 ± 0.54^b^	5.11 ± 0.31^a^

*Different superscript letters per row (a, b) indicate significant differences (P < 0.05) between groups*.

Regarding shedding, the percentage of samples positive to *C. abortus* by PCR in vaginal swabs after abortion or parturition was significantly (*P* < 0.05) high in control ewes compared to vaccinated and sentinel groups (Table 3). As a result, the amount of shedding of *C. abortus* the day of abortion or parturition was also significantly higher (*P* < 0.05) in control animals than in vaccinated and sentinel groups (Table 3).

**Figure 3:**
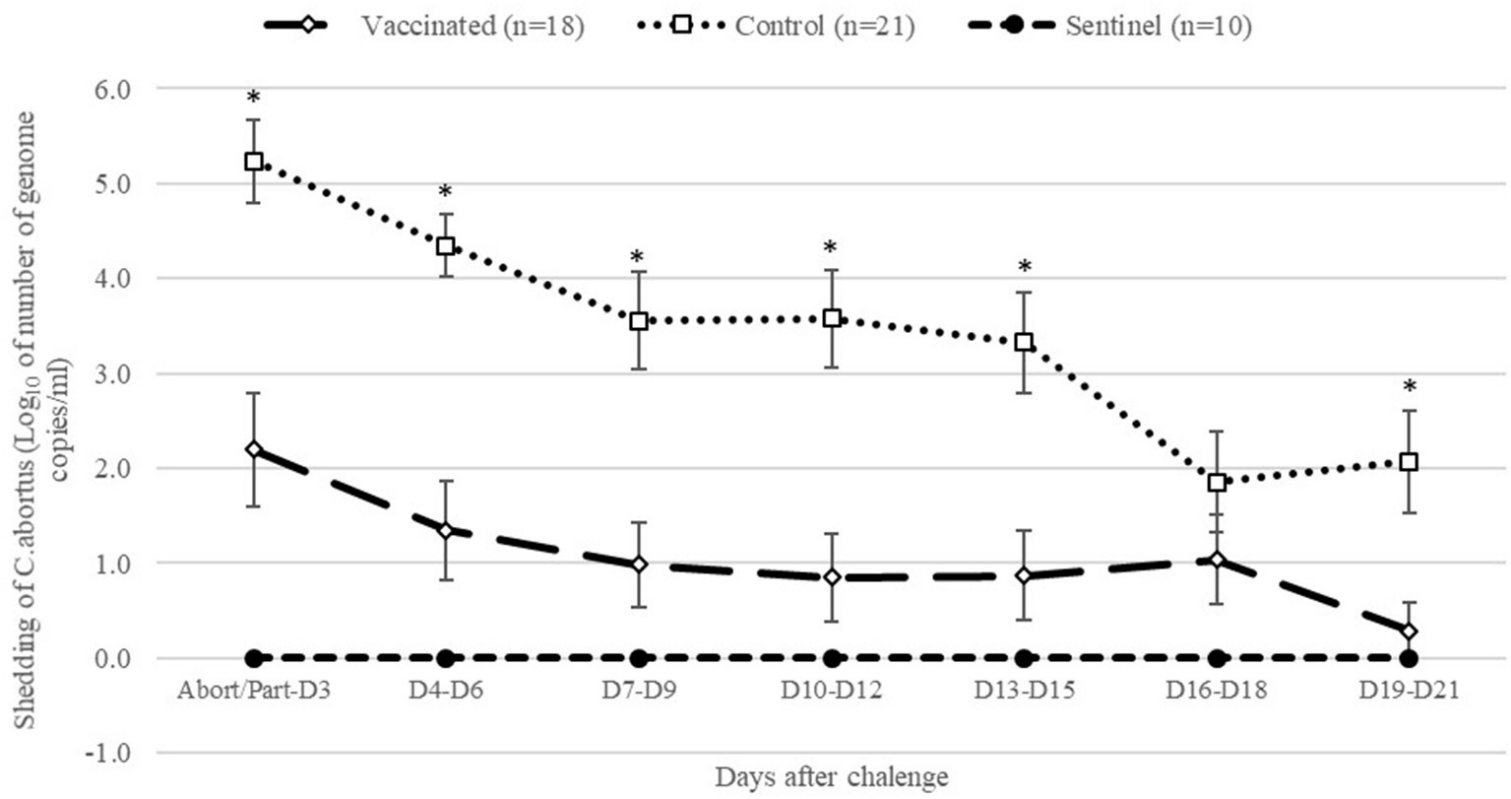
Geometric means of daily shedding (Log_10_ of number of genome copies of *C. abortus* per vaginal swab ± SEM) of control, vaccinated and sentinel group from abortion/parturition to 21 days after experimental infection. *Indicates significant differences (*P* < 0.05) between the control and other groups (vaccinated and sentinel).

After abortion or parturition, as shown in Figure 3, control ewes had significantly (*P* < 0.05) higher shedding of *C. abortus* than vaccinated and sentinel ewes from abortion/parturition to 15 days after and between 19 and 21 days after abortion/parturition. Overall, the AUC of total shedding from abortion or parturition to 21 days after was significantly (*P* < 0.05) greater in the control group than those of the vaccinated and sentinel groups (Table 3). Furthermore, the percentage of samples positive to *C. abortus* in vaginal swabs from abortion or parturition to 21 days after was significantly (*P* < 0.05) higher in control animals compared to vaccinated and sentinel ewes (Table 3).

#### Post-challenge Rectal Temperatures

**Figure 4:**
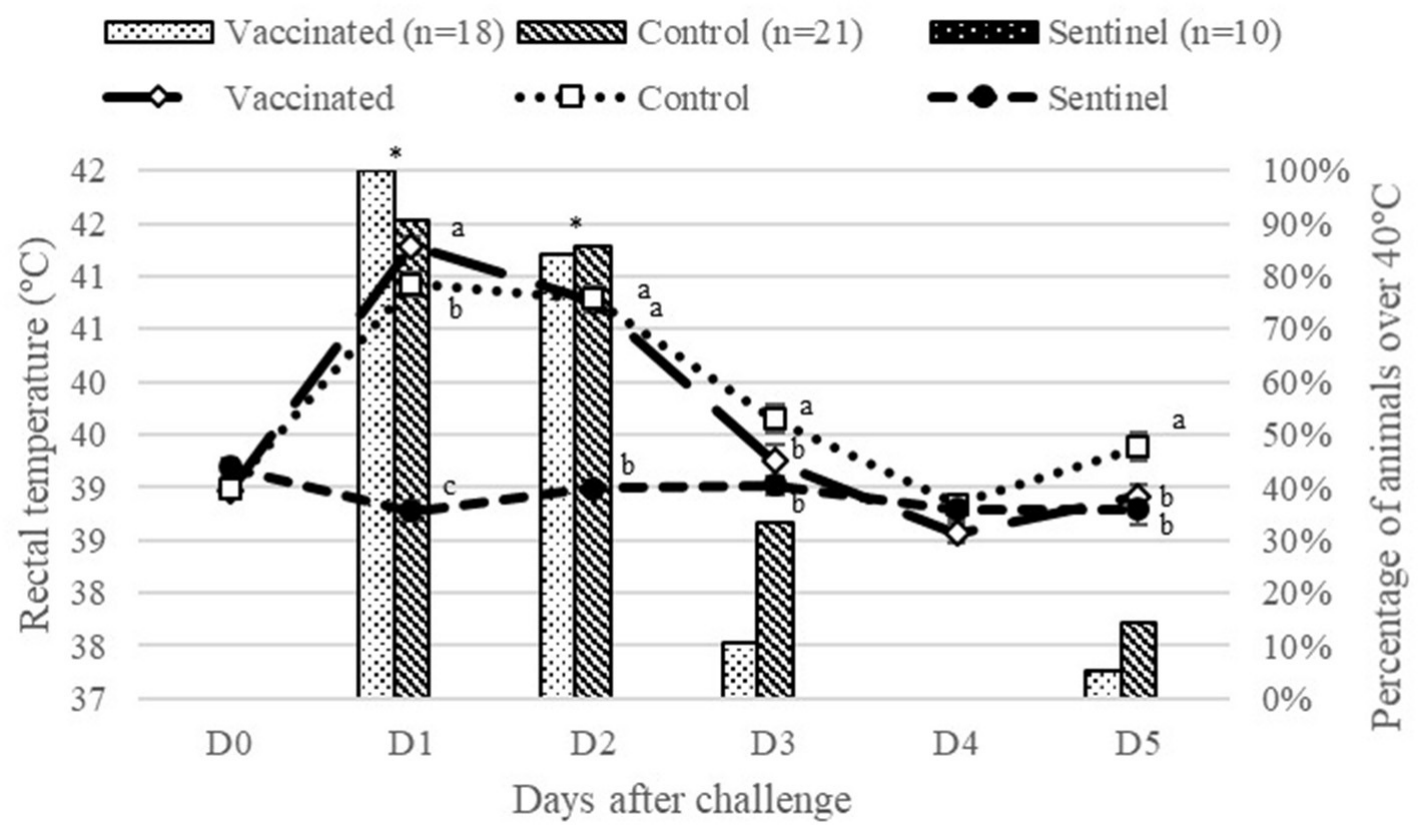
Average of rectal temperatures and percentage of animals with temperatures over 40°C from vaccinated and control groups from challenge to day 5 after experimental infection. Different superscript letters (*a, b, c*) indicate significant differences (*P* < 0.05) between groups in terms of rectal temperatures (°C) per day. *Indicates significant differences (*P* < 0.05) between infected groups (vaccinated and control) compared to the sentinel group in terms of percentage of animals over 40°C.

Rectal temperature values before challenge were similar in all groups. One day after challenge, the infected groups (vaccinated and control) presented an increase in average temperature, as shown in Figure 4. The control group had significantly (*P* < 0.05) higher rectal temperatures compared to the vaccinated and sentinel group. The vaccinated group had significantly higher rectal temperatures compared to the sentinel group (*P* < 0.05) 1 day after challenge. Afterwards, rectal temperatures decreased in these groups. Infected groups had significantly (*P* < 0.05) higher rectal temperatures than sentinel group 2 days after challenge. The percentage of animals with temperatures over 40°C in the infected groups was also significantly (*P* < 0.05) greater than the sentinel group (Figure 4). Three days after challenge, no significant differences were observed among vaccinated and sentinel groups in terms of average rectal temperature and percentage of animals over 40°C. However, the control group had a significantly (*P* < 0.05) higher average for rectal temperatures compared to the sentinel group (Figure 4). No significant differences were observed among all groups 4 days after challenge, but 5 days after challenge the average of rectal temperature of the control group was significantly (*P* < 0.05) higher than those of the vaccinated and sentinel groups.

#### Body Weight of Lambs

**Figure 5:**
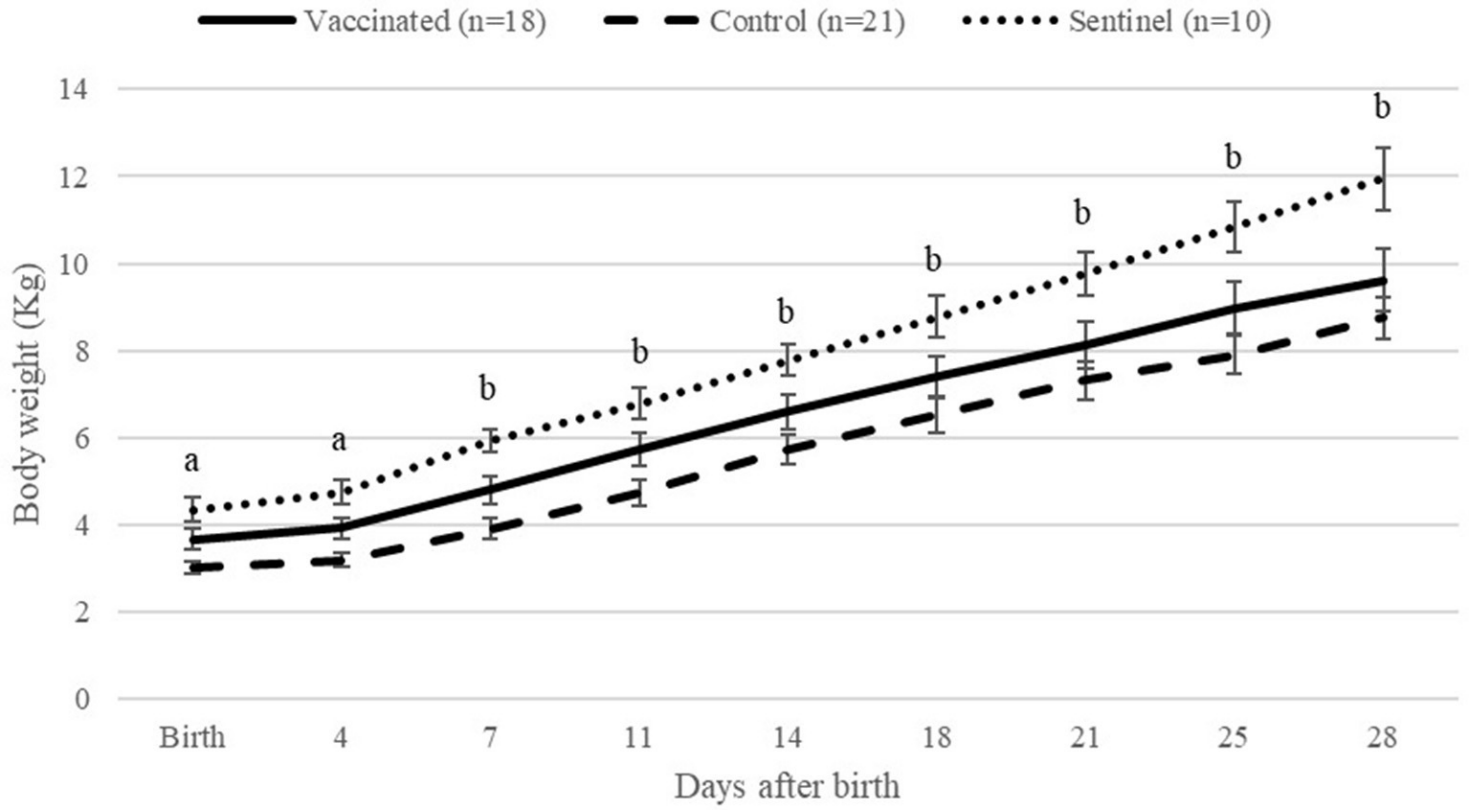
Average of lamb body weights per group from birth to 28 days after birth per day. ^a^Indicates significant differences (*P* < 0.05) between the control and other groups (vaccinated and sentinel). ^b^Indicates significant differences (*P* < 0.05) between the control and sentinel group.

The body weight of lambs from the control group was significantly (*P* < 0.05) lower than that of lambs from the vaccinated and sentinel groups at birth and 4 days after parturition (Figure 5). From 7 to 28 days after parturition, lambs from control ewes had significantly (*P* < 0.05) lower body weight than lambs from the sentinel group, but no differences were observed between the vaccinated and control groups. No significant differences were observed between lambs from the vaccinated group and sentinel group during the whole study.

## Discussion

OEA is one of the abortion diseases with the greatest economic impact worldwide, in terms of small ruminant production. One of the measures for chlamydial disease control is vaccination using commercial attenuated or inactivated vaccines. However, several authors (27, 36, 37) have questioned the efficacy of most of the current inactivated commercial vaccines. Regarding live vaccines, recently some researchers have warned about attenuated strain 1B, associating it with increase of OEA outbreaks in flocks vaccinated with this live vaccine (18, 19). For this reason, research continues to focus on a commercial product that must be effective, safe, and economically viable. The present study has shown that INMEVA, a recently commercialized inactivated vaccine developed against OEA, is effective in promoting a significant decrease in the number of reproductive failures and minimizing chlamydial shedding at delivery.

The effectiveness of a vaccine candidate may be linked to several factors such as the amount of antigen present, the inactivation method used, the choice of adjuvant(s), or the frequency of administration [reviewed in (38)]. Nevertheless, validating all these aspects in the relevant host using a pregnant sheep model would be excessively expensive and time-consuming. Due to this fact, mouse models have been considered as a useful economic tool to evaluate new vaccine candidates that prevent abortion and decrease chlamydial shedding sharply [reviewed in (29)]. Therefore, when developing the INMEVA vaccine, the choice of the inactivation method, the amount of antigen included in the formulation, as well as the adjuvant selected, were based on experimental mouse models (data not shown). Thus, experiment 1 shows the results of the INMEVA vaccine comparing them with the efficacy results obtained with an experimental inactivated vaccine widely used in different studies (i.e., the UMU vaccine). This UMU vaccine was previously tested on murine and ovine models, both with systemic (28, 36) and local (39–41) challenge, showing exceptionally good protective results in all cases. In these studies, the strain used to conduct these challenges was the same strain used to develop the UMU vaccine.

In the non- pregnant model, the UMU vaccine was more effective than the INMEVA vaccine, with respect to body weight or the presence of *C. abortus* in the liver. However, no significant differences were found between both vaccines in the pregnant model in terms of reproductive failure, pup number per mouse or the presence of *C. abortus* in the liver or uterus. The proven efficacy of both vaccines in the mice-pregnant model indicates that the antigen they contain confers the desired immunity response. The antigen of both vaccines tested were inactivated by an inactivating agent that acts on nucleic acids, but it preserved the integrity of the protein antigens. It has been reported that other chemicals, such as formaldehyde (27, 28, 42) and glutaraldehyde (10, 43) have been incorporated into the formulation of vaccines, both experimental and commercial, against OEA. But chemicals like these can destroy proteins, such as the polymorphic outer membrane proteins -POMPs- (44) and the oligomeric form of the MOMP (20) that may be essential to induce an adequate protective immune response; hence, the alteration of these proteins would decrease the effectiveness of this inactivated vaccine. In addition, a suitable inactivated vaccine must include an appropriate adjuvant that enhances an effective immune response. It has been shown that the adjuvant used in the formulation plays an important role in defining the immune response induced (cellular and/or humoral). Furthermore, it is known that a cellular immune response with relevant IFN-γ production is important in solving infection from *C. abortus* both in mice (45) and ovine (38). The adjuvant of INMEVA vaccine combines aluminum hydroxide and DEAE-dextran. Aluminum salts promotes formation of multimolecular aggregates, when it interacts with the antigen and these aggregates or multimolecular structure are more easily phagocytized (46). Moreover, DEAE-Dextran is commonly used to facilitate the infection of cell cultures *in vitro* (33) as it is a molecule that enhances the adhesion of Chlamydiae to target cells, which may increase the amount of antigen reaching the antigen presenting cells located in the immunization zone (46).

Once development in murine model was completed, the definitive validation of the efficacy of the vaccine must be performed in pregnant sheep models, since this ruminant species is the natural host of *C. abortus*. Thus, in experiment 2 the efficacy of the INMEVA vaccine was assessed comparing the reproductive failures and the bacterial shedding as well as the evaluation of the weight gain by the offspring, in relation to an unvaccinated infected control group. The experimental challenge caused a slight but significant increase in rectal temperature: 1°C for 2 consecutive days in both infected groups, as previously described (36, 37, 42, 47), showing that both groups were effectively challenged. Furthermore, the abortions and stillbirths suffered by the control non-vaccinated animals showed similar lesions, i.e., multifocal to coalescent suppurative placentitis with variable degree of severity, to those described in previous experimental studies on OEA (32). The results on reproductive performance and bacterial shedding showed that this new commercial vaccine, in addition to being safe, also provides a remarkable level of protection against an experimental challenge of *C. abortus* in pregnant sheep. These data are based on a 75% reduction in reproductive disorders and a 55% reduction in animals shedding *C. abortus* on the day of parturition/abortion when compared to the infected control group. In addition, the average amount of chlamydial shedding, measured in number of *C. abortus* genome copies, is about 1,000 times lower in vaccinated animals, not only on the day of parturition/abortion, but over the next 21 days when vaginal swabs are collected. This fact is essential because it could contribute to lessening the risk of transmission, as ewes and lambs become infected around parturition when large amounts of Chlamydiae are released, thereby favoring the infection of naïve animals, via ingestion or inhalation (2, 4). The protection results shown by the INMEVA vaccine are notably better than those reported by other commercial inactivated vaccines previously tested in similar pregnant ovine models, where the decrease in reproduction failures did not reach 10% in relation to infection control (11, 36, 37). Likewise, these commercial vaccines did not show a significant decrease in the bacterial shedding after parturition/abortion, and a positive effect from vaccination was observed on offspring growth in the vaccinated group compared with the control group (37). Although to a lesser extent, this effect on the body weight of lambs has also been observed in animals vaccinated with INMEVA, since lambs from the control group had significantly lower body weights than lambs from the vaccinated and sentinel groups in the first 4 days after birth.

In relation to the inactivated experimental vaccines against OEA, the UMU vaccine was used in this paper as a positive protection control in the murine model. The UMU vaccine was also tested in a previous study in the pregnant ovine model obtaining similar efficacy (72%) compared to INMEVA (75%), considering the reduction of reproductive failures in the vaccinated animals in relation to the infection controls (36). However, the antigen production method used in the UMU vaccine, with a partial purification of chlamydia (28), is difficult to apply at the industrial stage, and would considerably increase production costs. On the other hand, considering the efficacy of recombinant vaccines, in a very recent study carried out with an experimental vaccine that used a combination of recombinant MIP and CPAF from *C. abortus* (24), the experimental vaccine reached 50% efficacy, with a decrease in abortions in the vaccinated group in comparison to the infection control group. However, although vaccinated animals showed less shedding of *C. abortus* on the vaginal swab compared to the infected control group, this difference was not statistically significant. The current investigation is focused toward this type of subcellular vaccines by targeting proteins (48), but researchers indicate that further studies are needed to improve protection outcomes and achieve viable economic industrial production for marketing on the part of pharmaceutical companies.

## Conclusion

The results obtained in this study have shown that the INMEVA commercial inactivated vaccine is safe, showing a suitable level of protection against OEA related to a significant decrease in reproductive failures and in chlamydial shedding, thereby limiting spread of infection in the flock. In addition, this vaccine has minimized the weight loss of the lambs at birth and during the first week of life. In summary, this vaccine confers protection to animals challenged with *Chlamydia abortus* in the pregnant sheep model and mice models; thus, the results shown indicate that this commercial vaccine is adequate for controlling and preventing OEA. However, future studies are necessary to elucidate the type of protective immune response that INMEVA induces and how it is associated with its effectiveness.

## Data Availability Statement

The datasets generated for this study are available on request to the corresponding author.

## Ethics Statement

The study carried out on mice was reviewed and approved by the Bioethical Committee of the University of Murcia. The study carried out in ewes was approved by the local authorities (Junta de Castilla y León) after evaluation and recommendation by the CSIC Animal Experimentation Committee.

## Author Contributions

CM and MF: conceived and designed the experiments, analyzed and interpreted the data, drafted, and edited the manuscript. RM and MS: drafted and edited the manuscript. JB: performed the experiments on the sheep model and edited the manuscript. MC: performed the experiments on the mouse model, analyzed and interpreted the data, drafted, and edited the manuscript. NO: performed the experiments on the mouse model, analyzed, and interpreted the data. JS: conceived and designed the experiments, performed the experiments on the mouse model, analyzed and interpreted the data, drafted, and edited the manuscript.

## Conflict of Interest

CM, MF, RM, and MS are employees of Laboratorios Hipra S.A. The remaining authors declare that this study received funding from Laboratorios Hipra S.A. The funder had the following involvement with the study: experiments conception and design, analysis and interpretation of data, manuscript drafting.
